# Every Man His Own Dentist, &c.

**Published:** 1841-12

**Authors:** 


***Every Man his own Dentist. The art of preventing the loss of the teeth, familiarly explained; with a description of the siliceous pearl teeth which never wear out, change colour, nor become offensive. Also, of the Tooth Renovator; being a new and improved system of supplying deficiences without extraction, &c.*** By Joseph Scott, Surgeon Dentist. London, 1838, pp. 120, 8vo.

If any other evidence, than that which is to be found in the title of the above named work, was wanting to establish its character, it may be found on nearly every one of its pages. We can regard it in no other light than as an advertisement, as a fulsome self-eulogy—a laboured effort on the part of the author to bring himself professionally into notice. His own excellence as an operator, the superiority of his plan of treating the teeth, and that of the artificial teeth employed by him, constitute the burthen of his remarks. And as it would seem fearful that his own statements would not be believed, he bolsters them up with approbatory certificates of what he calls, his ***siliceous pearl*** teeth. Now he may for ought we know, manufacture good porcelain teeth, but that he is a scientific or skilful practitioner, the exposé which he gives of his plan of practice, evidences very conclusively to our mind that he is not. His work savours very strongly of empiricism from beginning to end. For example, in preparing a cavity in a tooth for filling, the following is his description of his manner of doing it.

"The points" says he, "to be attained in excavating a tooth, are, first, to preserve the edges of the enamel round the cavity; secondly, to hollow it out in such a manner that the ***stopping, when in the cavity, should assume, as much as possible, the shape of a cone, the broad end being downwards***; thirdly, great care should be taken that a thin portion of the bone substance of the tooth (*even if decayed*), should be left to support the enamel; otherwise, the points of crystallization will inevitably break in."

Now any one at all acquainted with, or skilled in the art of filling teeth, knows that a cavity thus prepared cannot be thoroughly and effectually filled. The article which he employs for stopping teeth, is a "composition cement," known only to himself. Nostrum-mongers, in any of the departments of medicine should be looked upon with suspicion. A practice which is incapable of bearing the test of rigid scrutiny, is not entitled to confidence, and yet it has ever been the case, that there were not wanting those who were always ready to patronize quackery. The ignorance of the author, however, of the present treatise, is so palpable, that it would be difficult for him to deceive any except the most credulous and unsuspecting.

For the amusement of our reader we will quote one or two other passages. "It not unfrequently happens," says he, "that front teeth (owing to their thinness or want of cavity) are not capable of being permanently stopped. In such cases the only plan is to remove the diseased part, as before stated, which may be done without danger of injury, and stopped with the anodyne cement until they are no longer unsightly, when a renovator may be worn, or the remainder of the crowns may be cut or filed off and grafted."

The renovator of which he speaks consists of a cap, porcelain we presume from the description given of it, fitted to the crowns of one or more teeth, as the case may require. The effects that would inevitably result from covering the teeth in this manner are too palpable to need explanation. A depository is thus at once formed for putrescent matter and morbid secretions, whereby the complete destruction of the enclosed organs must be speedily effected, and the breath rendered more or less offensive.

Again, in speaking of the treatment of caries, he remarks, "when gangrene or caries attack two adjoining teeth, the difficulty of stopping both may sometimes render it necessary to extract one to save the other."

We pity those who have had the misfortune to be his patients, and for humanity's sake, would fain hope, that he has been less successful in making dupes than many of the empirics with which the profession is infested. That his work should have passed through five editions, we can only account, by supposing that he has used it for the purpose for which it seems expressly intended; namely, as an advertisement. If the people in England can be imposed upon by such downright and barefaced charlatanism, we can only say, that it is high time they should institute some test of professional qualification, that will guard them against the impositions, to which they, as well as those of most other countries, are daily exposed.—*Balt. Ed.*

